# Influence of Root Canal Filling and Coronal Restoration Quality on Periapical Health: A Retrospective Observational Study

**DOI:** 10.1155/ijod/4108809

**Published:** 2026-05-22

**Authors:** Marie-Agnès Gasqui, Matthieu Perard, Cyril Villat, Franck Decup, Joséphine Kerguen, Jean Iwaz, Delphine Maucort-Boulch, Brigitte Grosgogeat, Marjorie Zanini

**Affiliations:** ^1^ Faculty of Dentistry, Université Claude Bernard, Lyon 1, Lyon, France, univ-lyon1.fr; ^2^ Laboratory of Multimaterials and Interfaces (UMR CNRS 5615), Villeurbanne, France; ^3^ Department of Dentistry, Lyon Hospital, Hospices Civils de Lyon, Lyon, France, chu-lyon.fr; ^4^ Faculty of Dentistry, University of Rennes, Rennes, France, univ-rennes1.fr; ^5^ Signal and Image Processing Laboratory (INSERM UMR 1099), Rennes, France; ^6^ Department of Dentistry, CHU Rennes, Rennes, France, chu-rennes.fr; ^7^ Faculty of Dentistry, University of Paris Cité, Paris, France, sorbonne.fr; ^8^ INSERM UMR 1333 Oral Health, Montrouge, France; ^9^ Department of Dentistry, Charles Foix Hospital (AP-HP, Assistance Publique - Hôpitaux de Paris), Ivry-sur-Seine, France; ^10^ Université Paris Cité and Université Sorbonne Paris Nord, Inserm, INRAE, Centre for Research in Epidemiology and Statistics, F-75004, Paris, France; ^11^ University of Lyon, Villeurbanne, France, univ-lyon1.fr; ^12^ Claude Bernard University Lyon 1, Lyon, France, univ-lyon1.fr; ^13^ CNRS UMR 5558 Laboratory of Biometrics and Evolutionary Biology, Biostatistics and Health Team, Villeurbanne, France; ^14^ Public Health Division, Department of Biostatistics and Bioinformatics, Lyon Hospital, Hospices Civils de Lyon, Lyon, France, chu-lyon.fr; ^15^ Department of Dentistry, Pitié Salpêtrière Hospital, Paris, France

**Keywords:** apical periodontitis, coronal restoration, cross-sectional study, dental radiography, root canal filling

## Abstract

**Objective:**

The aim of this study was to assess the association between the technical quality of root canal treatment and coronal restoration and periapical status in endodontically treated teeth and to explore their combined effects.

**Methods:**

This retrospective cross‐sectional observational study included 257 patients and 798 endodontically treated teeth. Coronal restoration quality was assessed both clinically, using FDI criteria, and radiographically and categorized as good or poor restoration. Root canal fillings were classified as adequate or inadequate (good endodontic treatment [GE] vs. poor endodontic treatment [PE]) based on length, density, and taper. Periapical status was evaluated using the periapical index (PAI). Associations between treatment quality parameters and periapical status were analyzed using odds ratios (ORs) with 95% confidence intervals (CIs).

**Results:**

Apical periodontitis (AP) was observed in 49.0% of root‐filled teeth. Inadequate coronal restoration, when clinical and radiographic criteria were combined, was significantly associated with AP (OR = 1.43; 95% CI [1.08–1.90]). Inadequate root canal treatment showed a stronger association with AP (OR = 2.30; 95% CI [1.64–3.21]), with all technical parameters (length, density, and taper) significantly influencing periapical status. Marginal adaptation alone, whether assessed clinically or radiographically, was not significantly associated with AP. When combined factors were analyzed, a gradient in risk was observed: teeth with inadequate root canal treatment (PE + GR and PE + PR) showed significantly higher odds of AP compared with the reference group (GE + GR), whereas adequate root canal treatment combined with poor restoration (GE + PR) was not significantly associated with AP.

**Conclusion:**

Both root canal treatment and coronal restoration quality are associated with periapical health. However, their effects differ, with root canal treatment quality showing a stronger association with periapical status. Importantly, an adequate coronal restoration alone does not appear sufficient to compensate for an inadequate root canal filling.

## 1. Introduction

Apical periodontitis (AP) is an inflammatory response to bacterial contamination of the endodontic system. In 2021, a review estimated the global prevalence of individuals with at least one AP to be 52% and reported that the incidence of AP was higher in root‐filled teeth than in untreated teeth (39% vs. 5%) [1]. Given this high prevalence and the known association of AP with various systemic diseases (e.g., rheumatoid arthritis and cardiovascular disease), further research into the risk factors associated with AP is needed.

The primary goal of an endodontic treatment is to prevent or cure AP by chemomechanical disinfection followed by system filling, including root filling and permanent coronal restoration [2]. A number of reports on the association between coronal leakage and bacterial contamination of root‐filled teeth have prompted numerous cross‐sectional studies that assessed the importance of quality coronal restoration and/or root canal filling on periapical health [3, 4]. Although most authors agree that good‐quality procedures are associated with a healthy periapical status, there is still conflicting evidence regarding the respective roles of root canal filling and coronal restoration quality, as well as their potential interaction in influencing periapical health.

Some authors have suggested that the quality of the restoration has a greater impact [5, 6], while others have emphasized the importance of the quality of the root canal filling [7–9]. Recent studies have also highlighted that the effect of inadequate root canal filling on periapical status may be exacerbated by poor coronal restoration quality, suggesting an interaction between these factors [10].

Furthermore, despite the large number of publications on this topic, a meta‐analysis conducted in 2011 led to inconclusive results and difficult comparisons due to a lack of standardization of study protocols and endpoints [11]. In addition, the relevance of the criteria used in cross‐sectional studies should be carefully examined because the quality of a root canal treatment can only be assessed radiographically, whereas the quality of a coronal restoration should be assessed clinically and radiographically. In 2011, Gillen et al. [11] stated that there are no clinical data to support the notion that the quality of a restoration can be adequately assessed by periapical radiographs alone. Indeed, radiographs are two‐dimensional representations of three‐dimensional structures that cannot reliably assess the quality of a coronal restoration [12, 13]. For example, a radiograph may readily identify a sealing defect at a proximal site but not an open margin at a buccal or lingual site. This has raised concerns about the relevance of radiographic criteria alone because they may lead to an overestimation of the quality of a coronal restoration. Therefore, cross‐sectional studies using both clinical and radiographical criteria are needed to assess coronal restoration and provide more accurate or reliable data for future meta‐analyses.

Finally, to the best of our knowledge, the last available French data on this topic were published more than a decade ago and derived only from monocentric studies carried out in hospitals [8, 14]. This is of particular concern given that cross‐sectional studies provide only a snapshot of a population at a specific time point and may not reflect current clinical practice. Over the past decade, significant advances in endodontic techniques and materials—including the widespread use of nickel–titanium instrumentation, improvements in root canal filling protocols, and developments in adhesive restorative systems—have substantially modified clinical practice and may have influenced treatment quality and outcomes. In parallel, several countries have recently updated their epidemiological data using improved and standardized methodologies, highlighting the importance of periodic reassessment. For instance, longitudinal comparisons conducted in German and Belgian populations over periods exceeding 20 years have demonstrated changes in periapical status and treatment quality over time [15–17]. In addition, assessing the prevalence of AP within a population provides valuable insights into treatment needs from a public health perspective and may help guide healthcare planning and resource allocation for the prevention and management of endodontic disease.

Moreover, recent large‐scale reviews on the prevalence of AP have included very limited French data, often relying on a single study, which further underscores the need for updated and methodologically robust investigations [1]. Periodic assessments of endodontic treatment quality within specific populations are essential to monitor temporal trends, identify treatment needs, and explore factors associated with periapical health. Such data also contribute to improving the comparability and reliability of future systematic reviews and meta‐analyses.

In this context, and to address the limitations of previously published French studies, the present multicenter observational study—conducted in both hospital and private practice settings—aimed to determine whether the quality of root canal treatment has a greater impact on periapical health than the quality of coronal restoration, using both clinical and radiographic evaluation criteria.

The null hypothesis of the study was that there is no association between the technical quality of root canal treatment or coronal restoration and periapical status and that the combined quality of these treatments does not influence periapical health.

## 2. Materials and Methods

The study was conducted in France between March 2019 and September 2020 by ReCOL (Recherche en Odontologie Clinique), the first French practice‐based research network.

All participants were given full information about the study and asked to give signed consents to participate.

The study protocol was approved by the local committee for ethics in research (Comite de Protection des Personnes Ile‐de‐France III; 3660‐NI 01/29/2019) and registered on ClinicalTrials.gov (NCT03854526) [18].

### 2.1. Patient Selection

As part of RESTO DATA, the present study considered the radiographs of 822 patients seeking routine (nonemergency) dental care. Between March 2019 and September 2020, any new patients were eligible patients and were enrolled consecutively without any selection criteria, provided they agreed to participate. These radiographs were collected by 76 investigators: 40 from 39 private practices and 36 from 13 hospital clinics located in 13 French cities (Lille, Brest, Rennes, Paris, Colombes, Ivry‐Sur‐Seine, Reims, Creteil, Clermont‐Ferrand, Bordeaux, Lyon, Toulouse, and Nice). During each visit, the investigators collected qualitative data on the conditions of all dental restorations present in each patient’s dentition.

Teeth that had undergone endodontic treatment (i.e., those with radio‐opaque material in the root canal) were first identified from panoramic radiographs and then examined using intraoral radiographs.

For this study, the inclusion criteria were patient consent to participate and the availability of high‐quality periapical radiographs. Patients were excluded from the study if they refused to participate, if their records contained missing clinical data, if they had radiographs of teeth with filling material in the pulp chamber only, if they had more than one unevaluable or unscorable parameter, or if their radiographs were of poor quality (with no use of a beamholder especially).

### 2.2. Clinical Evaluation

The clinical data collected encompassed various parameters, including the tooth position (anterior and posterior), the dental arch involved (maxillary or mandibular), the type of restoration (direct filling, partial coverage, total coverage, or lost or temporary restoration), and the quality of the coronal restoration.

The quality of the coronal restoration was clinically assessed according to FDI criteria 5 (fracture of material and retention) and 6 (marginal adaptation) [19]. Each criterion was evaluated at three distinct levels

• ideal restoration: score FDI 1 and 2 (clinically excellent, very good, and clinically good);
• minor deviation: score FDI 3 (clinically sufficient/satisfactory); and
• major deviation: score FDI 4 (clinically unsatisfactory but reparable) and/or 5 (clinically poor—replacement necessary).

In this study, restorations graded as “ideal restoration and minor deviation” were designated as “good restoration” (GR), and those with “major deviation” were designated as “poor restoration” (PR).

### 2.3. Radiographic Evaluation

The periapical radiographs were subjected to independent reviews by an experienced endodontist (author ZM) and a general dentist (author GMA). Prior to the study, 120 periapical radiographs (not included in the analysis) were used to calibrate the examiners for each parameter (quality and type of the coronal restoration, periapical health, and quality of the root canal treatment). The reproducibility of the graders’ scores was assessed using Cohen’s kappa statistics (for inter‐observer and intra‐observer agreement) at day 0, month 1, and month 6. The inter‐observer and intra‐observer kappa statistics demonstrated almost perfect agreement (>0.81) for each parameter [20].

For the present study, the investigators collected the following radiographic data: quality of the coronal restoration, quality of the root canal filling, presence and type of post (screw, metal, fiber, and temporary), and the periapical index (PAI) [21].

The quality of a coronal restoration was assessed radiographically on the basis of the radiographic appearance, in accordance with the criteria presented by Ray and Trope [5]. The restoration was considered good (GR) when the margins were intact and an adequate marginal sealing was applied. It was considered poor (PR) in the presence of recurrent caries and/or open margins or in the absence or loss of restoration.

The quality of an endodontic filling was assessed according to three parameters:

• the length of the root filling (“adequate” if the filling material was present in the root canal at ≤ 2 mm from the radiographic apex vs. “inadequate” if > 2 mm or if the filling material was present beyond the apical foramen);
• the density of the root filling (“adequate” if the root filling was homogeneous without visible voids vs. “inadequate” if voids were visible or in the case of a missed canal);
• the shape of the root canal filling (“adequate” if there was a regular increase in the transverse diameter from the apex to the canal entrance vs. “inadequate” if there was an irregular increase).

**Figure 1:**
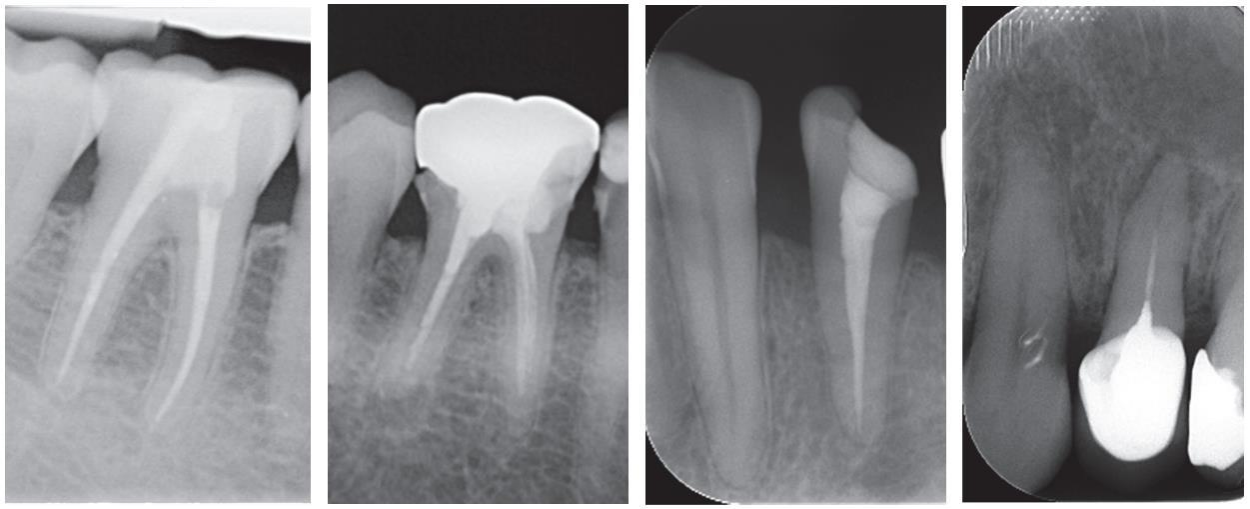
Examples of radiographs (from left to right). GE, good endodontic treatment; GR, good restoration; PE, poor endodontic treatment; and PR, poor restoration.

An endodontic treatment was considered good endodontics (GE) if the length of the filling material was adequate with no voids and had a consistent shape from the orifice to the apex. It was considered poor endodontics (PE) if at least one of the above parameters was inadequate (examples of GR, PR, GE, and PE are shown in Figure 1).

In multirooted teeth, each parameter was assessed separately for each root. In cases of overlapping roots, angulation radiographs (if available) were used to score each parameter. If a root could not be evaluated, the corresponding tooth was excluded from the analysis.

The final PAI score corresponded to the root with the highest PAI value, and the overall filling quality was determined by the root with the lowest quality score.

Regarding the periapical status, the Orstavik periapical indices were dichotomized in order to distinguish “healthy” from “diseased” (i.e., PAI 1 or 2 vs. PAI 3, 4, or 5).

### 2.4. Statistical Analyses

Categorical variables were described as numbers and percentages. The quality of endodontic treatment, its location, and the quality of the type of coronal restoration were described by the periapical status in bivariate analyses. χ^2^, Fisher tests, and odds ratios (ORs) with their 95% confidence intervals (95% CI) were calculated for each variable.

Logistic regressions were performed to explore the associations between the periapical status and the quality of coronal restoration (clinical criteria alone, radiographic criteria alone, and overall quality) and between the periapical status and the quality of endodontic treatment. ORs with their 95% CIs were calculated, and a Wald χ^2^ test was performed for each variable.

All the tests were two tailed and differences were considered statistically significant at *p*‐values of less than 0.05. All statistical analyses were performed using R.4.3.2 software (R Foundation for Statistical Computing, Vienna, Austria).

The study complied with the STROBE guidelines [22].

## 3. Results

### 3.1. Study Population and Characteristics of Root‐Filled Teeth

**Figure 2:**
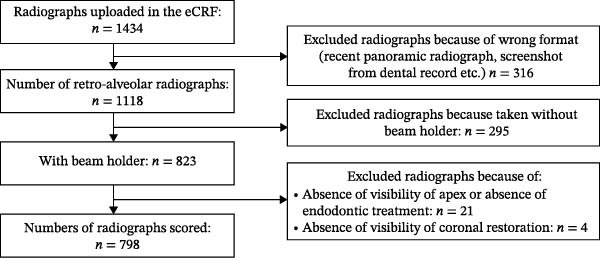
Flowchart of radiographs included in the study.

The present study included 257 patients and examined 798 teeth (Figure 2 and Table 1). The mean age of the patients was 51 years (min: 18; max: 82). Females, new patients, and intermediate professions were the most common subgroups (64.2%, 44.7%, and 27.6%, respectively). The majority of root‐filled teeth were posterior teeth or located in the maxillary arch (67.0% and 60.0%, respectively).

**Table 1:** Characteristics of the 257 included participants and the 798 endodontically treated teeth.

Characteristic	*n* (%)
Age	
18–29 years	31 (12%)
30–59 years	139 (54%)
≥ 60 years	87 (34%)
Sex	
Males	92 (35.8%)
Females	165 (64.2%)
Occupation/Socio‐professional class	
Craftsmen, traders, and business leaders	45 (17.5%)
Managers, higher intellectual professions	12 (4.7%)
Intermediate professions	71 (27.6%)
Employees	4 (1.6%)
Workers	84 (32.6%)
Retirees	16 (6.2%)
Not in active employment	25 (9.8%)
Reason for dental visit	
Routine checkup	146 (56.8%)
Toothache	39 (15.2%)
Carious lesion	7 (2.7%)
Failure of a dental restoration	6 (2.4%)
Failure of a dental prosthesis	17 (6.7%)
Periodontal disorder	21 (8.1%)
Other	21 (8.1%)
Dental arch involved	
Maxillary	479 (60.0%)
Mandibular	319 (40.0%)
Tooth location	
Anterior	263 (33.0%)
Posterior	535 (67.0%)

### 3.2. Coronal Restoration and Periapical Status

The prevalence of AP in endodontically treated teeth was 49.0%. An overview of the tooth types, their location, and their coronal status in relation to the periapical condition is shown in Table 2. Neither the arch location nor the tooth type showed a significant association with periapical disease. Most restorations consisted of full coverage (79.2%), and this treatment was significantly associated with a “healthy” periapical status (OR = 0.66, 95% CI [0.45–0.96]). Furthermore, neither the use nor the type of post was significantly associated with a “diseased” periapical status (*p* = 0.677 and 0.1262, respectively).

**Table 2:** Associations of the main coronal restoration characteristics with periapical health.

Characteristic	Total	Healthy periapical status	Diseased periapical status	*p*‐Value	OR (95% CI)
Dental arch involved	—	—	—	0.8507	—
Maxillary	479	243 (50.7%)	236 (49.3%)	—	1
Mandibular	319	164 (51.4%)	155 (48.6%)	—	0.97 (0.73–1.29)
Tooth location	—	—	—	0.1017	—
Anterior	263	145 (55.1%)	118 (44.9%)	—	1
Posterior	535	262 (49.0%)	273 (51.0%)	—	1.28 (0.95–1.72)
Restoration type	—	—	—	0.09457	—
Direct filling + partial coverage	134	57 (42.5%)	77 (57.5%)	—	1
Full coverage	632	334 (52.8%)	298 (47.2%)	—	0.66 (0.45–0.96)
Temporary or lost	32	16 (50.0%)	16 (50.0%)	—	0.74 (0.34–1.61)
Post‐retained restoration	—	—	—	0.677	—
Absent	280	140 (50.0%)	140 (50.0%)	—	1
Present	518	267 (51.5%)	251 (48.5%)	—	0.94 (0.70–1.26)
Post type^a^	—	—	—	0.1262 ^∗^	—
Screw post	103	48 (46.6%)	55 (53.4%)	—	—
Metal post	404	216 (53.5%)	188 (46.5%)	—	—
Fiber post	6	3 (50.0%)	3 (50.0%)	—	—
Temporary	5	0 (0.0%)	5 (100%)	—	—
None	280	140 (50.0%)	140 (50.0%)	—	—
Radiographical quality of the restoration	—	—	—	0.06345	—
Adequate (good restoration)	579	307 (53.0%)	272 (47.0%)	—	1
Inadequate (poor restoration)	219	100 (45.7%)	119 (54.3%)	—	1.34 (0.98–1.83)
Clinical quality of the restoration	—	—	—	0.2844	—
Adequate (good restoration)	623	324 (52.0%)	299 (48.0%)	—	1
Inadequate (poor restoration)	175	83 (47.4%)	92 (52.6%)	—	1.20 (0.86–1.68)
Overall quality of the restoration	—	—	—	0.01923	—
Adequate (good restoration)	482	262 (54.4%)	220 (45.6%)	—	1
Inadequate (poor restoration)	316	145 (45.9%)	171 (54.1%)	—	1.43 (1.08–1.90)

^a^Odds ratios were not calculated because the use of a post was not found to be significantly associated with diseased periapical status.

^∗^Use of Fisher instead of chi‐2 test.

When the coronal restorations were clinically scored, 78.0% (623/798) were GRs, of which 48.0% showed a diseased periapical status. When the coronal restorations were scored radiographically, 72.5% (579/798) were GRs, of which 47.0% showed a diseased periapical status. No association with AP was found when the clinical and radiographic criteria were considered independently (OR = 1.20, 95% CI [0.86–1.68] and OR = 1.34, 95% CI [0.98–1.83], respectively). However, when the clinical and radiographic criteria were combined, PR (39.5%, 316/798) was found to be significantly associated with a diseased periapical status (*p* = 0.019, OR = 1.43, 95% CI [1.08–1.90]) (Table 2).

### 3.3. Root Canal Treatment and Periapical Status

Table 3 shows data on the quality of root canal treatment and its association with periapical status. An analysis of the endodontic treatments shows that 53.6% had adequate shapes, 49.7% had adequate densities, and 52.5% had adequate lengths. Overall, 26.7% (213/798) of the root‐filled teeth had GE, of which 140 (65.7%) were considered “healthy.”

**Table 3:** Associations of the main canal filling characteristics with periapical health.

Characteristic	Total	Healthy periapical status	Diseased periapical status	*p*‐Value	OR (95% CI)
*Shape* (*taper*)	—	—	—	0.00205	—
Adequate	428	240 (56.1%)	188 (43.9%)	—	1
Inadequate	370	167 (45.1%)	203 (54.9%)	—	1.55 (1.17–2.05)
*Density*	—	—	—	<0.001	—
Adequate	397	237 (59.7%)	160 (40.3%)	—	1
Inadequate	401	170 (42.4%)	231 (57.6%)	—	2.01 (1.52–2.67)
*Length*	—	—	—	<0.001	—
Adequate	419	249 (59.4%)	170 (40.6%)	—	1
Inadequate (overall)	379	158 (41.7%)	221 (58.3%)	—	2.05 (1.54–2.72)
Underfilled	290	122 (42.1%)	168 (57.8%)	—	2.02 (1.49–2.74)
Overfilled	89	36 (40.4%)	53 (59.6%)	—	2.16 (1.36–3.46)
*Overall quality*	—	—	—	<0.001	—
Good	213	140 (65.7%)	73 (34.3%)	—	1
Poor	585	267 (45.6%)	318 (54.4%)	—	2.30 (1.64–3.21)

*Note*: The *p*‐values were calculated from chi‐2 tests.

Three parameters significantly influenced the periapical status: the density of the root filling (*p* = 0.002; OR = 1.55, 95% CI [1.17–2.05]), the shape of the root filling (*p* < 0.001; OR = 2.01, 95% CI [1.52–2.67]), and the length of the root filling (*p* < 0.001; OR = 2.05, 95% CI [1.54–2.72]).

Looking at the overall quality, PE was significantly associated with periapical disease (OR = 2.30, 95% CI [1.64–3.21]).

### 3.4. Quality of Coronal Restoration and Endodontic Treatment Combined

The associations between the overall quality of coronal and endodontic treatment and the periapical status are shown in Table 4. A risk gradient was observed: compared with the reference combination “GE + GR,” the odds of a diseased periapical area tended to be slightly increased with “PR + GE,” significantly increased with “PE + GR,” and strongly increased with “PE + PR.” Only the combinations “PE + GR” and “PE + PR” were statistically associated with a diseased periapical status (OR = 2.35, 95% CI [1.56–3.58] and OR = 3.07, 95% CI [1.99–4.79], respectively), whereas the combination “GE + PR” was not found to be significantly associated with AP. Furthermore, assessing coronal restoration clinically only, radiographically only, or clinically and radiographically did not alter the associations with the periapical status.

**Table 4:** Associations of the endodontic and restoration quality combinations with the periapical health.

Criteria for restoration‐quality assessment	Total	Healthy periapical status	Diseased periapical status	OR (95% CI)	*p*‐Value
Radiographic and clinical	—	—	—	—	<0.0001
GE + GR	141	97	44 (31.2%)	1	—
GE + PR	72	43	29 (40.3%)	1.49 (0.82–2.68)	—
PE + GR	341	165	176 (51.2%)	2.35 (1.56–3.58)	—
PE + PR	244	102	142 (58.2%)	3.07 (1.99–4.79)	—
Radiographic only	—	—	—	—	<0.0001
GE + GR	171	117	54 (31.6%)	1	—
GE + PR	42	23	19 (45.2%)	1.79 (0.89–3.56)	—
PE + GR	408	190	218 (53.4%)	2.49 (1.71–3.64)	—
PE + PR	177	77	100 (56.5%)	2.81 (1.82–4.38)	—
Clinical only	—	—	—	—	<0.0001
GE + GR	167	112	55 (32.9%)	1	—
GE + PR	46	28	18 (39.1%)	1.31 (0.66–2.56)	—
PE + GR	456	212	244 (53.5%)	2.34 (1.62–3.42)	—
PE + PR	129	55	74 (57.4%)	2.74 (1.71–4.43)	—

*Note*: The *p*‐values were calculated from chi‐2 tests.

Abbreviations: GE, good endodontics; GR, good restoration; PE, poor endodontics; PR, poor restoration.

## 4. Discussion

The aim of this study was to determine whether the quality of a root canal treatment had a more positive effect on periapical health status than the quality of a coronal restoration, or vice versa. It was found that diseased periapices were more frequently seen after inadequate root canal treatments than after inadequate coronal restorations. When factor combinations were considered (with GE + GR as a reference), only the combinations “PE + GR” and “PE + PR” were significantly associated with periapical disease. Although the CIs did not allow a definitive conclusion in favor of the “GE + PR” combination, these results suggest that inadequate coronal restoration alone was not significantly associated with periapical disease. Overall, this indicates that the quality of the endodontic filling may have a stronger influence on periapical health. These results align with previous studies, which generally report that the technical quality of root canal treatment shows the strongest association with periapical status [6–8, 10, 23].

A root canal filling of adequate technical quality—defined by appropriate length, density, and taper—is more likely to reflect properly performed instrumentation and irrigation procedures. Effective chemomechanical disinfection achieved during shaping reduces the intracanal microbial load, a key determinant of periapical healing. In addition, a well‐compacted root canal filling may act as a physical barrier, “entombing” residual bacteria and preventing their proliferation, thereby creating a biological environment conducive to periapical healing [2]. Each poor‐quality criterion (length, density, or taper) was significantly associated with the presence of periapical lesions, with taper irregularities particularly important, as they may reflect procedural errors that leave necrotic tissue or microorganisms. Voids in root canal filling may also facilitate rapid recontamination of the root canal system.

Coronal restorations function as a secondary barrier, preventing bacterial ingress into the root canal system. The influence of coronal leakage depends on multiple factors, including the extent of leakage, presence or absence of restoration, duration of bacterial challenge, quality of the root canal filling, and number and virulence of invading microorganisms [24]. In the present study, inadequate coronal restorations alone were not significantly associated with periapical disease; however, when evaluated using combined clinical and radiographic criteria, adequate restorations were significantly associated with a healthy periapex. Full‐coverage restorations were the only restoration type significantly linked to periapical health, consistent with their known advantages in preserving tooth structure and long‐term survival. Although inadequate restorations alone were not significantly associated with periapical disease in this study, their loss of integrity can allow bacterial penetration and contamination of the root filling over time [24].

Our findings are consistent with international evidence. Kielbassa et al. [10] reported in a large Austrian subpopulation that both the quality of root canal obturation and postendodontic restoration were associated with periapical health, and inadequate fillings significantly increased the odds of periradicular disease. These results confirm that while root canal treatment quality is a primary determinant of periapical status, coronal restoration quality remains an important contributing factor. Comparable findings have been reported in other countries, including Morocco, Finland, and Sweden, highlighting the need for up‐to‐date, region‐specific data [25–27].

The prevalence of AP in root‐filled teeth was 49%, higher than reported in other studies (33% in Hommez et al. [12] and 40.9% in Song et al. [28]), which may be related to the lower rate of adequate endodontic treatment—precisely, 26.7% in this study, compared to 34.4% in Hommez et al. [12] study and 35.6% in that of Song et al. [28]. These results seem discouraging but must be interpreted in the context of general dental practice (i.e., a context where material and/or time constraints are generally greater than in specialist or university clinics).

Previous studies have reported lower technical quality of root canal treatments performed in general dental practice compared with those carried out by specialists or within academia [29–32]. This gradient in treatment quality may reflect differences in clinical experience, training, and access to advanced technologies.

Several factors may contribute to inadequate root canal treatment. Undetected root canals and insufficient instrumentation are among the most frequently reported causes. The former may be partly explained by the limited use of magnification tools in general practice. A recent study by Nosrat et al. [33] reported that only 1% of general dental practitioners routinely use optical aids, compared with 67% of endodontists (*p*  < 0.001). Magnifications improve not only canal detection but also canal negotiation and overall treatment quality.

In addition, the choice of obturation technique may influence the technical quality of root canal fillings. General practitioners more frequently use simplified techniques such as the single‐cone technique [33], which is less time‐consuming but has been associated with a higher risk of voids within the filling material. These voids, which are not always detectable radiographically, may compromise the density and sealing ability of the obturation [34]. Earlier studies have also shown that the use of the single‐cone technique tends to increase with practitioner age [35, 36]. From an educational perspective, continuing professional development plays a key role in maintaining and improving clinical skills settings [30–32]. However, the structure and content of continuing education programs in France may vary, and participation does not always involve hands‐on training. This variability may contribute to differences in the level of endodontic competence among practitioners. Therefore, structured continuing education programs combining theoretical updates with practical training throughout the professional career appear essential to support both knowledge updating and skill development in endodontics.

In addition to operator‐related factors, case complexity appears to play a key role in the technical quality of root canal treatment. A recent prospective study from the PREDICT network [37] showed that several preoperative difficulty factors—such as canal visibility, calcifications, or root curvature—were significantly associated with intraoperative complications. Moreover, despite managing more complex cases, endodontists reported fewer complications than general practitioners, highlighting differences in expertise and case management. These findings suggest that variability in treatment quality may partly reflect differences in case difficulty assessment, clinical decision‐making, and the ability to manage complex anatomical situations in routine practice.

While the real‐life design of the present study is a strength, as it reflects routine clinical practice, it also entails variability in practitioner experience and decision‐making, which should be considered when interpreting the results. Beyond technical limitations, behavioral and organizational factors also play a role. Dahlström et al. [38] reported that Swedish general dental practitioners often accept suboptimal root canal filling quality, following a pragmatic approach summarized as “it’s good enough”. Such attitudes may reflect clinical time constraints, perceived patient tolerance, or a prioritization of other aspects of treatment. These findings highlight that inadequate root canal treatment is not only a matter of knowledge or skill but also of clinical decision‐making and professional judgment in real‐world practice.

In addition to reflecting the realities of everyday endodontic practice, the present results are in line with those of other studies [7, 9, 13] and with the percentage of adequate treatment found in the French population in 2002 (21%) [14] and 2009 (27%) [8]. These observations suggest that, despite advances in materials and techniques, the overall quality of root canal treatment in general practice may not have substantially improved over time. This is further supported by a recent 22‐year follow‐up cross‐sectional study conducted in a Belgian population, which reported no significant improvement in the technical quality of root canal treatments over time [15].

In terms of quality criteria, root canal fillings were evaluated according to the length, density, and taper criteria commonly used in France [39]. Irregularities or inconsistencies in the taper (such as ledges, perforations, or apical transport) may indicate biomechanical procedural failures or operator errors that are likely to interfere with a proper mechanical cleaning of the root canal walls and leave residual necrotic tissue or microorganisms—the main etiological agents of periapical lesions. Indeed, Santos et al. [40] found that altered taper was significantly associated with periapical lesions.

Root canal disinfection is primarily based on chemomechanical disinfection, and canal shaping is a critical aspect. Indeed, shaping ensures that endodontic files clean the canal walls and effectively remove necrotic debris and bacteria. Increasing the root canal taper allows better apical penetration of the irrigation needle and improves the exchange of sodium hypochlorite solution [41]. The taper also enables a tight three‐dimensional obturation [2, 42]. In this study, each poor‐quality criterion alone (shape, density, or length of the root canal filling) was statistically significantly associated with the presence of a periapical lesion. In particular, shape should be an important criterion in assessing the quality of root filling.

This study evaluated the quality of the restoration both clinically and radiologically, which is rather unusual in cross‐sectional studies on this topic [6, 12, 28, 43]. According to the FDI criteria, the quality of a coronal restoration should be assessed by checking for inadequate sealing (i.e., clinical or radiological evidence of gaps between the tooth wall and the restorative material) [19, 44]. The present study found that 27.5% of coronal restorations were inadequate by radiographic criteria, 21.9% by clinical criteria, and 39.6% by both criteria. These results indicate that assessing the quality of a restoration using radiographic criteria alone may lead to an underestimation of quality. In addition, neither clinical nor radiographic criteria alone were found to be significantly associated with a diseased apex (*p* = 0.2844 and *p* = 0.06345, respectively). However, when those criteria were combined, adequate restorations were significantly associated with a healthy periapex (*p* = 0.01923). These results support the importance of using both clinical and radiographic criteria to reliably assess the quality of coronal restorations, which is consistent with previous recommendations by Craveiro et al. [45].

In this study, full coverage was the only restoration type found to be significantly associated with a healthy periapical region. This is very interesting, as there is still a lack of evidence in the literature regarding the influence of the type of coronal restoration on the periapical status [46]. Currently, there is no consensus on the best restorative material for endodontic treatment; however, the predominance of full coverage in this study (79.2%) suggests that this method is preferred over direct filling because it is associated with less substance loss. In addition, a previous study reported that practitioners prefer crowns [47], especially in the posterior region, because of the better 5‐year and 10‐year survival rates of these indirect restorations compared with direct restorations [48, 49].

The present study did not find a statistical association between “temporary or lost restorations” and periapical status, probably because of lack of statistical power due to the small sample size (only 32 out of 798). This calls for a more specific study on the influence of the type of restoration on periapical health.

Furthermore, in this study, particular attention was paid to the representativeness of the patient sample and the relevance of the assessment criteria. Unlike other monocentric studies with similar designs, the data were collected from a wide range of hospitals and private practices in different regions of France. This broad scope increases the generalizability of the results.

Despite the findings made, this study has several limitations. To assess periapical lesions and root canal treatment quality, several recent studies used cone‐beam computed tomography (CBCT) [45, 50–54] to overcome two conventional X‐ray limitations: the two‐dimensional representation of a three‐dimensional structure [55] and confusion of periapical lesions with lesions limited to cancellous bone [56]. Despite the superiority of CBCT [57], the current recommendations do not yet favor its systematic use (AAE and AAOMR Joint Position Statement 2015) [58] because of the “as low as reasonably achievable” principle. In fact, a source with a small “field of view” exposes the patient to 10–100 times the radiation dose received with standard periapical radiography [59]. Therefore, retroalveolar radiography remains the radiographic modality of choice, especially in routine care. CBCT is certainly more accurate than radiographs in detecting missed canals, but the latter could be suspected on traditional radiographs due to the absence of filling material in a root or off‐centered filling in the case of several canals in the same root. Precautions were taken when reading the teeth, especially the mesiobuccal roots (where missed canals are often found) [60]. In addition, the readings were taken by a specialist in endodontics who was trained in the quality of root canal readings.

Cross‐sectional studies are often criticized for their limited ability to reflect the dynamics of most biological processes, which would make them almost unsuitable for evaluating treatment procedures. A periapical lesion seen on radiographs may be a developing lesion, a lesion in the process of healing, or a mass of scar tissue [61]. Nevertheless, Petersson et al. [62] in 1991 and Hugoson et al. [63] in 1995 found that the number of periapical lesions healed after 10 years was equal to the number of newly developed lesions, suggesting that cross‐sectional studies may be reliable in assessing the long‐term success of endodontic treatments [62, 63]. In addition, cross‐sectional studies are generally less susceptible to investigator bias than longitudinal studies and provide important facts about the distribution, the prevalence, and the determinants of endodontic diseases. Finally, cross‐sectional studies may not answer all questions but help to identify potential risk factors in high‐level clinical trials.

Finally, in this study, the extraction criteria were not fully stratified. In accordance with the ESE and the FDI guidelines, it is desirable for future prospective studies to be conducted using data stratified by variables such as the type of tooth (indicative of the degree of technical difficulty), previous apical status, preoperative pulpal status, initial versus secondary treatment, delay since last treatment, time elapsed before coronal seal, type of restoration (direct/indirect), and intraoperative factors. Such studies will highly clarify the importance of each variable in the quality of endodontic treatment.

## 5. Conclusion

This cross‐sectional study shows that both the quality of root canal treatment and the quality of coronal restoration are significantly associated with periapical status in endodontically treated teeth.

Within the limits of this study design, the technical quality of root canal treatment appears to have a stronger association with periapical health. However, optimal outcomes are achieved when both high‐quality endodontic treatment and adequate coronal restoration are present, as coronal restoration provides an additional protective effect by preventing reinfection of the root canal system.

A part of this article has previously been published as a preprint (Gasqui et al. [64]).

## Author Contributions

**Marie-Agnès Gasqui**: conceptualization, formal analysis, investigation, data curation, writing – original draft, writing – review and editing. **Matthieu Perard**: conceptualization, methodology, formal analysis, investigation, writing – original draft, writing – review and editing. **Cyril Villat**: conceptualization, methodology, formal analysis, data curation, writing – original draft. **Franck Decup**: conceptualization, methodology, formal analysis, investigation, data curation, writing – review and editing, project administration. **Joséphine Kerguen**: data curation, writing – original draft. **Jean Iwaz**: formal analysis, writing – review and editing, visualization. **Delphine Maucort-Boulch**: conceptualization, methodology, formal analysis. **Brigitte Grosgogeat**: conceptualization, methodology, data curation, formal analysis, writing – original draft, writing – review and editing, supervision, project administration. **Marjorie Zanini**: methodology, formal analysis, writing – original draft, writing – review and editing.

## Funding

This research did not receive any specific grant from funding agencies in the public, commercial, or not‐for‐profit sectors.

## Disclosure

All the above‐mentioned authors have revised and approved the submitted version of the manuscript.

## Conflicts of Interest

The authors declare no conflicts of interest.

## Data Availability

The data that support the findings of this study are available from the corresponding author upon reasonable request.
